# Dorsal clock networks drive temperature preference rhythms in *Drosophila*

**DOI:** 10.1016/j.celrep.2022.110668

**Published:** 2022-04-12

**Authors:** Shyh-Chi Chen, Xin Tang, Tadahiro Goda, Yujiro Umezaki, Abigail C. Riley, Manabu Sekiguchi, Taishi Yoshii, Fumika N. Hamada

**Affiliations:** 1 Division of Developmental Biology, Cincinnati Children’s Hospital Medical Center, Cincinnati, OH 45229, USA; 2 Graduate School of Natural Science and Technology, Okayama University, Okayama 700-8530, Japan; 3 Department of Neurobiology, Physiology and Behavior, University of California, Davis, Davis, CA 95616, USA; 4 These authors contributed equally; 5 Lead contact

## Abstract

Animals display a body temperature rhythm (BTR). Little is known about the mechanisms by which a rhythmic pattern of BTR is regulated and how body temperature is set at different times of the day. As small ectotherms, *Drosophila* exhibit a daily temperature preference rhythm (TPR), which generates BTR. Here, we demonstrate dorsal clock networks that play essential roles in TPR. Dorsal neurons 2 (DN2s) are the main clock for TPR. We find that DN2s and posterior DN1s (DN1ps) contact and the extent of contacts increases during the day and that the silencing of DN2s or DN1ps leads to a lower temperature preference. The data suggest that temporal control of the microcircuit from DN2s to DN1ps contributes to TPR regulation. We also identify anterior DN1s (DN1as) as another important clock for TPR. Thus, we show that the DN networks predominantly control TPR and determine both a rhythmic pattern and preferred temperatures.

## In brief

The body temperature rhythm (BTR) is vital for maintaining homeostasis. *Drosophila* exhibit a daily temperature preference rhythm (TPR), which generates BTR. Chen et al. show that dorsal clock neurons in the brain form a time-dependent network and govern TPR by regulating a rhythmic pattern and a temperature setpoint.

## Graphical Abstract

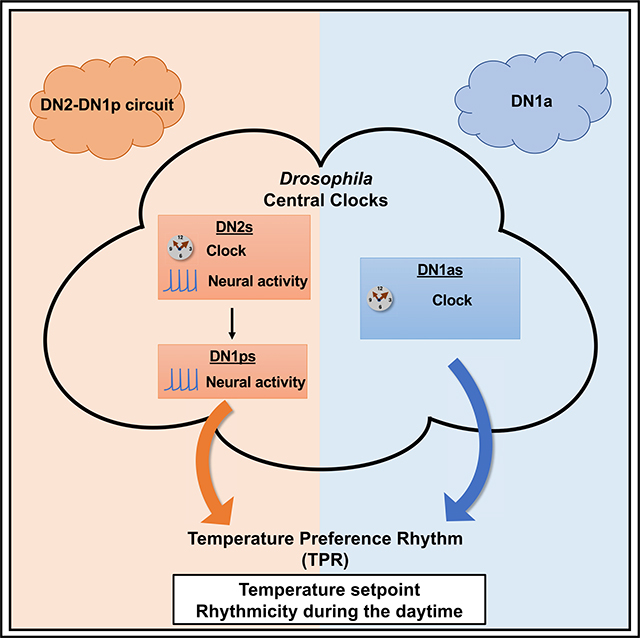

## INTRODUCTION

The body temperature rhythm (BTR) is fundamental for maintaining homeostasis, such as metabolic rates and sleep (Aschoff, 1983; Krauchi, 2002; Refinetti and Menaker, 1992; Weinert, 2010). An animal’s body temperature increases during wakefulness and decreases during sleep (Buhr et al., 2010; Duffy et al., 1998; Gilbert et al., 2004; Krauchi, 2007a, b; Lack et al., 2008; Morf and Schibler, 2013; Refinetti and Menaker, 1992). Several studies suggest that the mechanisms controlling BTR and locomotor activity rhythms are dissociated. Human body temperature still fluctuates when locomotor activity is restricted (Gander et al., 1986; Smith, 1969). Spontaneous internal desynchronization suggests that locomotor activity rhythms and BTR are experimentally dissociated (Lavie, 2001). Locomotor activity rhythms and the BTR are controlled by different output pathways from the suprachiasmatic nucleus (Saper et al., 2005); however, the underlying mechanisms controlling BTR remain largely unclear. At least two factors determine BTR. One is the pattern of body temperature change for 24 h. The other is the setting of the body temperature. Little is known about how BTR fluctuations are determined and how the temperature is set at a specific time of day.

While mammals regulate the BTR by generating or losing heat, *Drosophila* maintain a BTR via a temperature preference rhythm (TPR) (Goda and Hamada, 2019; Kaneko et al., 2012). Flies seek a colder temperature in the morning and the warmest temperature in the evening before nighttime sleep and then seek a colder environment during the night (Kaneko et al., 2012). As they are small ectotherms, the flies’ body temperature is close to the ambient temperature (Giraldo et al., 2019; Stevenson, 1985a, 1985b). Therefore, flies produce BTR by selecting a preferred environmental temperature (Goda and Hamada, 2019).

The fly brain contains approximately 150 clock neurons. Based on their locations and their cell sizes, clock neurons are divided into groups of lateral neurons (LNs) and dorsal neurons (DNs: DN1s [anterior DN1s (DN1as), posterior DN1s (DN1ps)], DN2s, and DN3s) (Allada and Chung, 2010). LNvs are the well-characterized main oscillators of locomotor activity rhythms, but not of the TPR. On the other hand, DN2s are the main clock neurons responsible for the TPR, but not for locomotor activity rhythms (Kaneko et al., 2012). Therefore, the main oscillators controlling locomotor activity rhythms and the TPR are dissociated. Importantly, we also determined that in both flies and mice, a G-protein-coupled receptor, calcitonin receptor regulates the BTR but not locomotor activity rhythms (Goda et al., 2018).

To understand the mechanisms by which TPR is controlled, we focused on the neural network in DNs with temperature-sensing pathways. Recent data suggest that DN1as and DN1ps receive temperature inputs. DN1as are the downstream target of cold-processing neurons (Alpert et al., 2020; Marin et al., 2020). DN1ps regulate temperature entrainment and are influenced by ambient temperature cues via chordotonal organs (Chen et al., 2015; Lamaze and Stanewsky, 2019; Zhang et al., 2010b). Both excitation and inhibition of DN1ps were observed during decreases and increases in the ambient temperature, respectively (Yadlapalli et al., 2018). Furthermore, DN1ps receive the inputs from the warm-sensing anterior cell neurons (ACs) (Hamada et al., 2008; Jin et al., 2021). Thus, we hypothesized that DN networks play important roles in TPR and sought to understand the relationship within DNs for TPR regulation.

Our data suggest that DN2s build a dynamic neural network with DN1ps, forming time-dependent interactions, contributing to controlling the TPR. The silencing of DN2s or DN1ps leads to a lower temperature preference, suggesting their roles in setting the preferred temperature. We also identified DN1as as another important clock neuron for TPR. Thus, we determined that the DN networks govern the TPR by generating the rhythmic pattern and selecting the preferred temperature setpoint.

## RESULTS

### DN1ps are involved with TPR regulation

**Figure 1.**
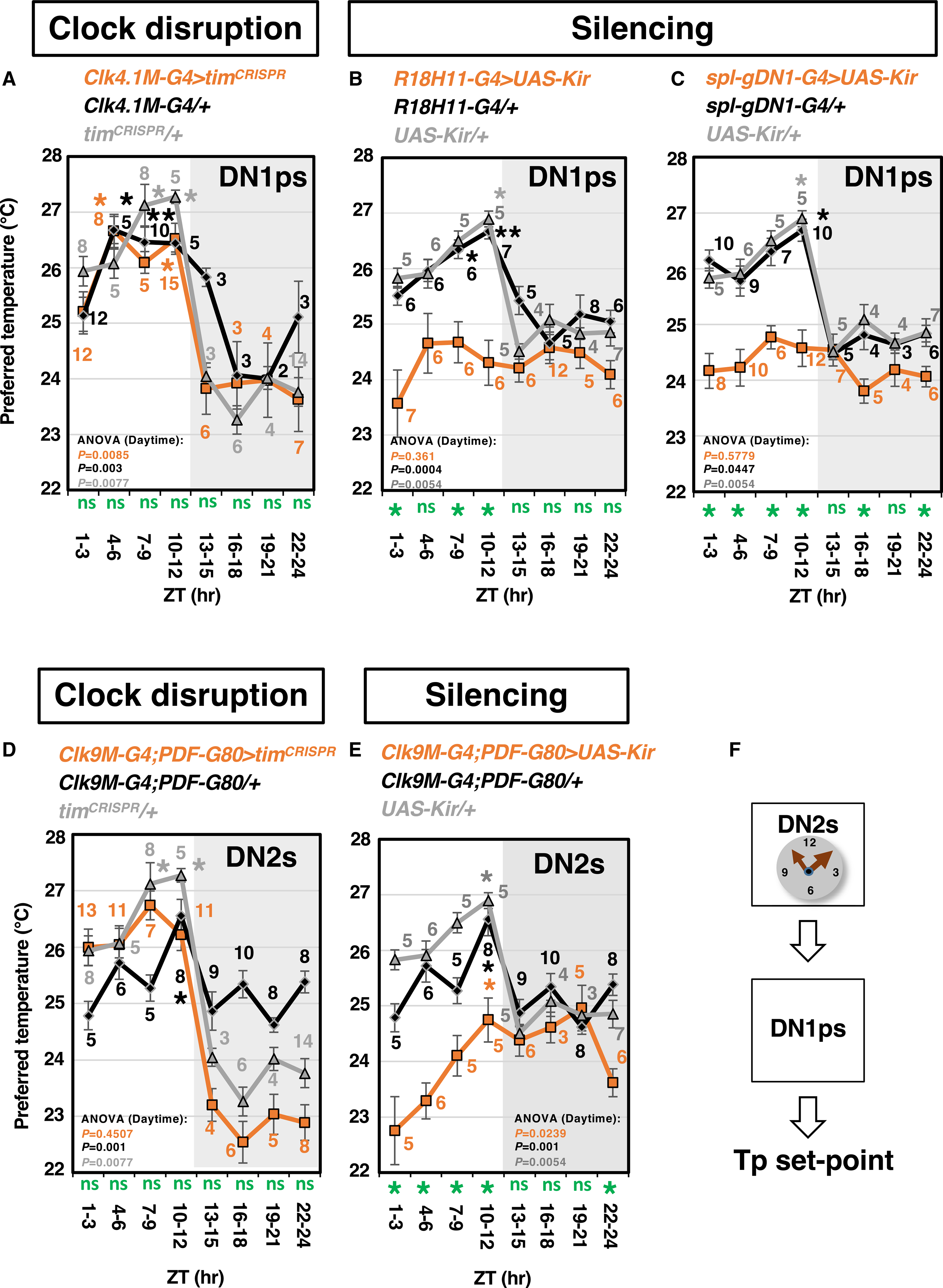
DN2s, but not DN1ps, are the main clock neurons for the daytime TPR (A–E) Comparison of the TPR between the flies with clock disruption or silencing (orange) and the control flies (*Gal4* [black] and *UAS* [gray]). Clock disruption by the expression of *tim*^*CRISPR*^ in DN1ps (A) and DN2s (D). Neuronal silencing using *UAS-Kir* with *R18H11-Gal4* (DN1p-specific) (B), *spl-gDN1-Gal4* (DN1p-specific) (C), and *Clk9M-G4;PDF-G80* (DN2s-specific) (E). (F) Schematic showing that DN2s and DN1ps are likely in the same pathway for TPR regulation. The numbers in the graphs represent the number of assays. The daytime TPR data were analyzed using one-way ANOVA and Tukey’s honestly significant difference post hoc test, and the data were compared with the Tp at ZT 1–3 (Table S1): **p < 0.01 and *p < 0.05. The Tp in experimental and control flies was compared at each time point using one-way ANOVA and Tukey’s honestly significant difference post hoc test: green *p < 0.05 for both controls and ns refers to not significant, which are shown below the x axis (Table S2).

To determine whether DNs play important roles in TPR, we disrupted the circadian molecular clock by employing CRISPR-Cas9 genome editing technique. A *timeless (tim)*-CRISPR line was recently developed and used to eliminate the circadian clock in each clock neuron group (Delventhal et al., 2019; Port and Bullock, 2016; Schlichting et al., 2019). First, we employed *tim*^*CRISPR*^ in all clock neurons and tested the TPR (Figure S1A). The preferred temperature (Tp) of both *tim-Gal4/+* and *tim*^*CRISPR*^/+ control flies showed the time-dependent Tp increase during the daytime, but the *tim* knockout in all clock neurons (*tim-Gal4*>*tim*^*CRISPR*^) abolished it (Figure S1A; Table S1). However, unexpectedly, DN1p clock-disrupted flies (*Clk4.1M-Gal4>tim*^*CRISPR*^) showed a significant time-dependent Tp increase during the daytime as controls (*Clk4.1M-Gal4/+* and *tim*^*CRISPR*^/+) (Figure 1A; Table S1). The data suggest that the functional clock in DN1ps is not required for TPR and that DN1ps are not the main clock neurons for the TPR in the daytime.

To determine whether the neural activity of DN1ps was involved in TPR, we silenced a subset of DN1ps by expressing the inward rectifying K^+^ channel Kir2.1 (*UAS-Kir2.1*). Because the expression of *UAS-Kir2.1* under *Clk4.1M-Gal4* caused lethality, we used *R18H11-Gal4* instead, which expresses seven to eight DN1ps (Guo et al., 2016). The Tp in DN1p-silenced flies lost a time-dependent Tp increase during the daytime and failed to show a Tp decrease during the day-to-night transition (from Zeitgeber time [ZT] 10–12 to ZT 13–15; ZT 0 represents light on) (Figure 1B; Table S1). Notably, the Tp in the control flies (the *R18H11-Gal4/+* and *UAS-Kir*/+) was around 25.5 to 26°C at ZT 1–3. However, the Tp in DN1p-silenced flies was around 23.5°C at ZT 1–3 and was significantly lower than that in controls during the daytime. The green stars were added at the bottom of the graphs to indicate when the Tp of experimental flies (orange) was significantly different from those of controls (black and gray) (Figure 1; Table S2).

To confirm the phenotype of DN1p silencing in TPR, we used another *Gal4* line, *spl-gDN1-Gal4*, which is more specifically expressed in five to six glutamatergic DN1ps. DN1p-silenced flies showed a severely abnormal TPR (Figure 1C) similar to that of *R18H11-Gal4>UAS-Kir* flies (Figure 1B). Thus, the neural activity, but not the clock, of DN1ps contributes to TPR regulation in terms of setting the Tp and the fluctuation of the TPR during the daytime and at the transition from day to night. The data suggest that the role of DN1ps in TPR is likely the downstream of the main clocks.

### DN1ps and DN2s are likely in the same circuit

Because DN2s are the main clock for the TPR (Kaneko et al., 2012), we employed *tim*^*CRISPR*^ using the DN2 driver, *Clk9M-Gal4;PDF-Gal80* and reevaluated TPR (Figure 1D). DN2 clock-disrupted flies lost the time-dependent Tp increase during the daytime but still showed a significant Tp drop at the day-to-night transition (Figure 1D; Table S1). We performed immunostaining using an anti-TIM antibody and verified the TIM deletion in DN2s (Figures S2A and S2B). For another control experiment, we used *tim-*RNAi to knock down *tim* in DN2s, which caused a similar phenotype as the DN2 clock-disrupted flies (Figure S1B; Table S1). Thus, the clock in DN2s plays an important role in regulating the time-dependent Tp increase during the daytime.

Next, we tested if the neural activity in DN2s is responsible for TPR. We silenced DN2s by expressing *UAS-Kir2.1* and then tested TPR. While the Tp in the control flies was around 25 to 26°C at ZT 1–3, the Tp in DN2-silenced flies was around 23°C (Figure 1E). Similar to the DN1p-silenced flies (Figures 1B and 1C), the DN2-silenced flies also showed significantly lower Tp than controls during the daytime (green star at the bottom of the graph) (Figure 1E; Table S2). Because DN1p or DN2 inhibition caused a lower Tp, the neural activity of DN1ps or DN2s contributes to setting the Tp. Given that the clock in DN2s, but not DN1ps, is important for TPR regulation, we hypothesized that DN2s might function upstream of DN1ps (Figure 1F).

### Neural contacts between DN2s and DN1ps

**Figure 2.**
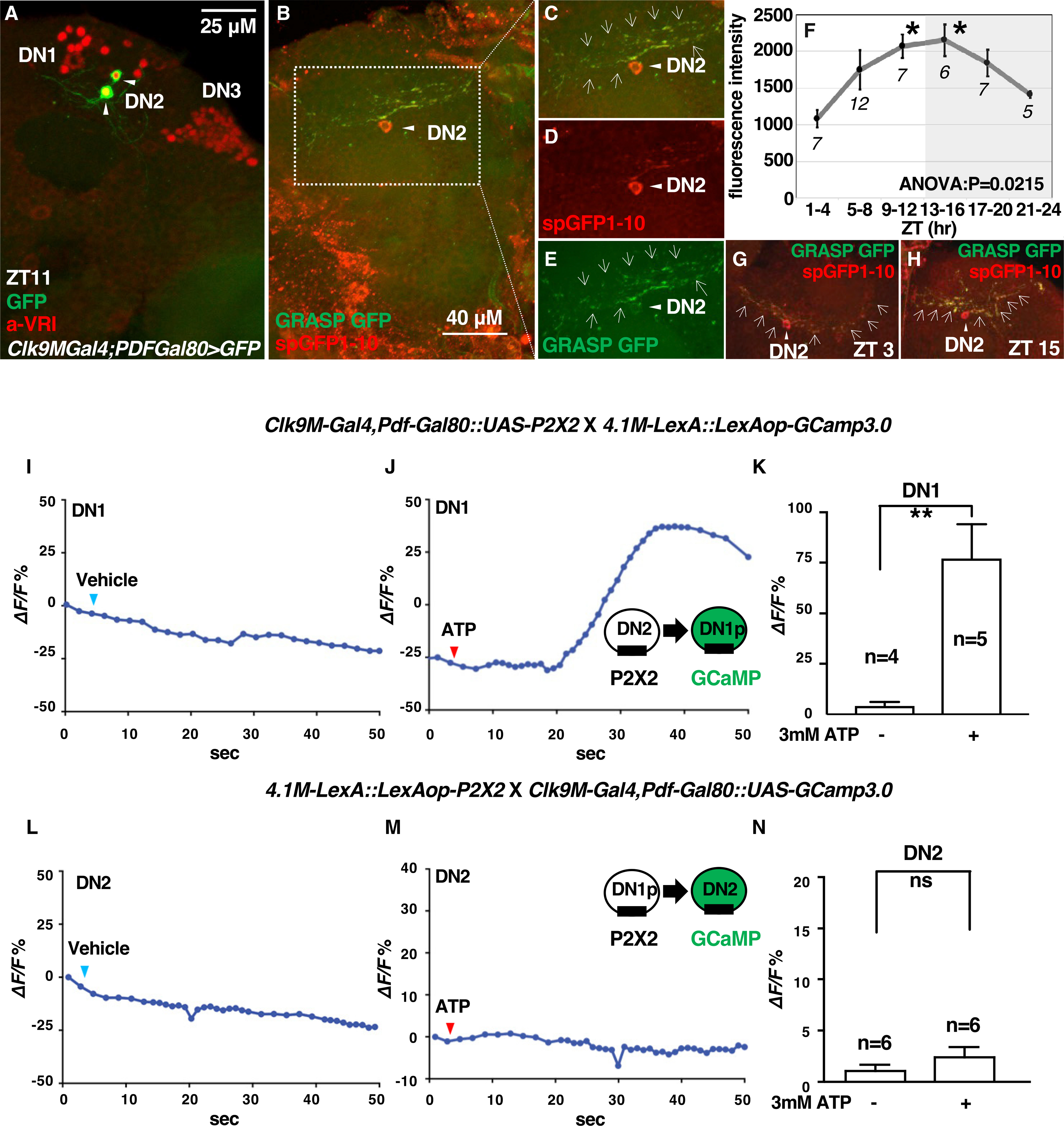
DN2s contact DN1ps (A) Representative image of the *Clk9M-Gal4/UAS-mCD8:GFP;PDF-Gal80/+* brain at ZT 11. DNs were immunostained by an anti-VRILLE (VRI) antibody (red). GFP is selectively expressed in DN2s (green; shown in arrowheads). (B–E) Representative image of the GRASP signals. The reconstituted GFP signals (GRASP GFP; green; shown as arrows in C and E) were detected (B, magnified in C–E). The soma and projection of DN2s (red) were stained with an anti-GFP antibody against spGFP1–10 expressed from a DN2 driver (D). (F–H) The GRASP signals were measured as the GFP fluorescence intensity and compared throughout the day (F). The numbers in the graphs represent the number of GRASP experiments. One-way ANOVA and Tukey-Kramer post hoc tests were used to compare the GFP fluorescence intensity at different times with that at ZT 1–4 (Table S3). Representative images of GRASP signals at ZT 3 (G) and ZT 15 (H). More and brighter contacts showed by the arrows were observed between DN2s and DN1ps at ZT 15 than at ZT 3. (I and J) Representative graphs of DN1p activation via P2X2 expression in DN2s. GCaMP3.0 and P2X2 were expressed in DN1ps and DN2s, respectively. The representative trace of GCaMP fluorescence in DN1ps after the bath application of 3 mM ATP (J) or the vehicle control (I). (L and M) Representative graphs of DN2 activity induced by P2X2 expression in DN1ps. GCaMP3.0 and P2X2 were expressed in DN2s and DN1ps, respectively. The representative trace of GCaMP fluorescence in DN2s through the bath application of 3 mM ATP (M) or the vehicle control (L). (K, N) The bar graphs show the mean maximum increases in GCaMP fluorescence in DN1ps (K) and DN2s (N) after the bath application of ATP or vehicle control. An unpaired *t*-test was used. The numbers in the graphs represent the number of experiments, and the experiments were performed from ZT 5–12.

DN2s and DN1ps are closely located in the dorsal part of the brain. We asked whether DN2s and DN1ps form local contacts in this region (Figure 2A). To this end, we performed a GFP Reconstitution Across Synaptic Partners (GRASP) experiment (Feinberg et al., 2008; Gordon and Scott, 2009; Kim et al., 2012). Although neither split-GFP fragment fluoresces itself, split-GFP fragments can reconstitute fluorescence upon contact (Gordon and Scott, 2009). We used a DN2 driver and a DN1p driver (*Clk4.1M-LexA*) to express one of the split-GFP fragments, *UAS-CD4:spGFP1–10* in DN2s and *LexAop-CD4:spGFP11* in DN1ps. We observed green fluorescence signals that represent the reconstituted GFP (GRASP GFP) in the flies expressing both split-GFP fragments (Figure 2E). We also labeled DN2s by a specific GFP antibody that recognizes the split-GFP1–10 fragment with the Cy5 staining (red) (Figure 2D), which colocalized with the GRASP signals (Figures 2B and 2C). As controls, we showed that the split-GFP1–10 fragment was only detected by the specific GFP antibody (Figure S3A). These data suggest that DN2s and DN1ps physically interact with one another.

### The extent of DN2-DN1p interactions increases during the daytime and peaks at dusk to the early night

We previously identified the microcircuit from sLNvs and DN2s and showed that its contacts fluctuate and peak before dawn (ZT 22–24) (Tang et al., 2017). Therefore, we asked whether the DN2-DN1p contacts also fluctuate and examined the extent of DN2 and DN1p GRASP contacts over a 24-h period. Strikingly, we found the brighter, broader, and more GRASP signals at ZT 15 (green in Figure 2H) along the process of DN2s (red in Figure 2H) compared with those at ZT 3 (Figure 2G). The fluorescence intensity of DN2-DN1p contacts increased from the morning (ZT 0) to evening (ZT 12) and peaked from dusk (ZT 9–12) to early night (ZT 13–16) (Figure 2F; Table S3). The data suggest that amounts of DN2 and DN1p GRASP contacts fluctuate over a 24-h period.

Since each split-GFP1–10 and split-GFP11 fragment was expressed by *Clk-Gal4s* (or LexA), the GFP moieties might fluctuate, which could drive the fluctuation of the overall GRASP signal for 24 h. However, we observed that split-GFP1–10 and split-GFP11 fragments did not show significant changes between ZT 0 and ZT 12 (Figures S3B and S3C). Thus, our data indicate that the extent of DN2 and DN1p GRASP contacts fluctuates over a 24-h period.

### DN2s activate DN1ps

To determine whether there is a functional relationship between DN2s and DN1ps and identify which neurons are upstream or downstream, we performed calcium imaging using GCaMP3.0 with the mammalian ATP-gated ionotropic P2X2 receptor (Tian et al., 2009; Yao et al., 2012). We excited upstream neurons by ectopically expressed P2X2 receptor through the bath application of ATP and determined whether the exogenous depolarization of the upstream neurons leads to a change in cytoplasmic calcium signaling in the downstream neurons. First, to test whether DN2s excite DN1ps, P2X2 was expressed in DN2s using *Clk9M-Gal4;PDF-Gal80* and GCaMP3.0 was expressed in DN1ps using *Clk4.1M-LexA*. The bath application of 3 mM ATP increased the GCaMP fluorescence in DN1ps (Figure 2J). The average increase over the baseline (ΔF/F) in DN1ps in response to ATP was significantly higher than that found with the vehicle controls (Figures 2I and 2K). The lack of response to the vehicle control confirmed that the increases in GCaMP fluorescence were specific to the activation of DN2s by ATP. As a control, we also used flies lacking P2X2 expression in DN2s, which did not show a response to ATP (Figure S3D). Based on these data, the excitation of DN2s by P2X2 and the subsequent depolarization of DN1ps led to an increase in the cytoplasmic calcium level in DN1ps (Figure 2K).

We also investigated whether DN1ps excite DN2s. P2X2 was expressed in DN1ps using *Clk4.1M-LexA*, and GCaMP3.0 was expressed in DN2s using *Clk9M-Gal4;PDF-Gal80* (Figures 2L–2N). However, the bath application of 3 mM ATP did not increase the GCaMP fluorescence in DN2s (Figure 2M), suggesting that DN1ps do not excite DN2s. The GRASP experiments showed dynamic contacts between DN2s and DN1ps (Figure 2F), suggesting that DN2s can modulate the neural activity of DN1ps in a time-dependent manner. Thus, our findings suggest that the DN2-DN1p microcircuit likely regulates the TPR.

### Hyperexcitation of DN2s caused a severe abnormal TPR during the daytime

Whereas DN2 clock-disrupted flies lost the time-dependent Tp increase during the daytime (Figure 1D), DN2-silenced flies still exhibited the time-dependent Tp increase with a lower Tp (Figure 1E). Due to the discrepancy in the phenotypes between DN2 clock-disrupted and DN2-silenced flies, we wondered whether DN2 clock disruption might lead to activation rather than inhibition of DN2s, and asked whether DN2 activation caused a severe abnormal TPR phenotype.

**Figure 3.**
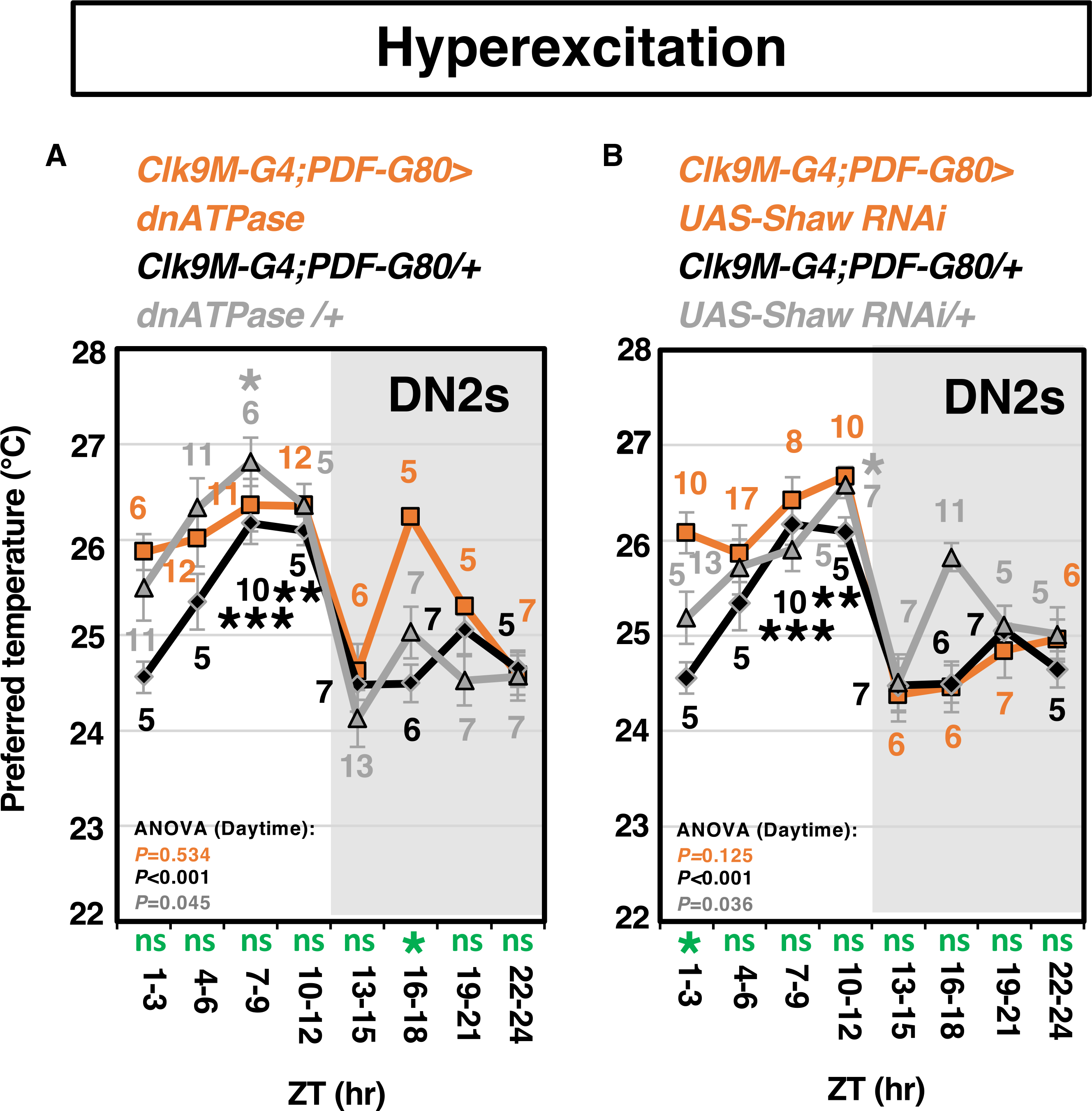
DN2 hyperactivation causes the abnormal daytime TPR TPR in DN2-hyperexcited flies. Hyperexcitation in DN2s was induced by the expression of *UAS-dnATPase* (A) and *UAS-Shaw-RNAi* (B) using *Clk9M-G4;PDF-G80* (Table S1). The Tp at each ZT was compared with those at ZT 1–3 using one-way ANOVA and Tukey’s honestly significant difference post hoc test.

The expressions of dominant-negative Na^+^/K^+^ATPase (dnATPase) or RNAi of Shaw potassium channels (Shaw-RNAi) induce neuronal hyperexcitation (Parisky et al., 2008). We expressed either dnATPase or Shaw-RNAi in DN2s and tested the TPR. Both DN2-hyperexcited flies failed to show the time-dependent Tp increase during the daytime (Figure 3; Table S1). Notably, DN2-hyperexcited flies (Figure 3) and DN2 clock-disrupted flies (Figure 1D) showed a similar TPR phenotype during the daytime, except DN2-hyperexcited flies with dnATPase or Shaw-RNAi showed a higher Tp compared with controls only at ZT16–18 or ZT 1–3, respectively (green star in Figures 3A and 3B and Table S2). It suggests that clock disruption in DN2s could lead to hyperactivation rather than inhibition in DN2s. Thus, our data indicate crucial roles for the neural activity of DN2s in TPR fluctuations, and the clock likely modulates DN2 activity.

### DN1as are another important neuron for TPR

**Figure 4.**
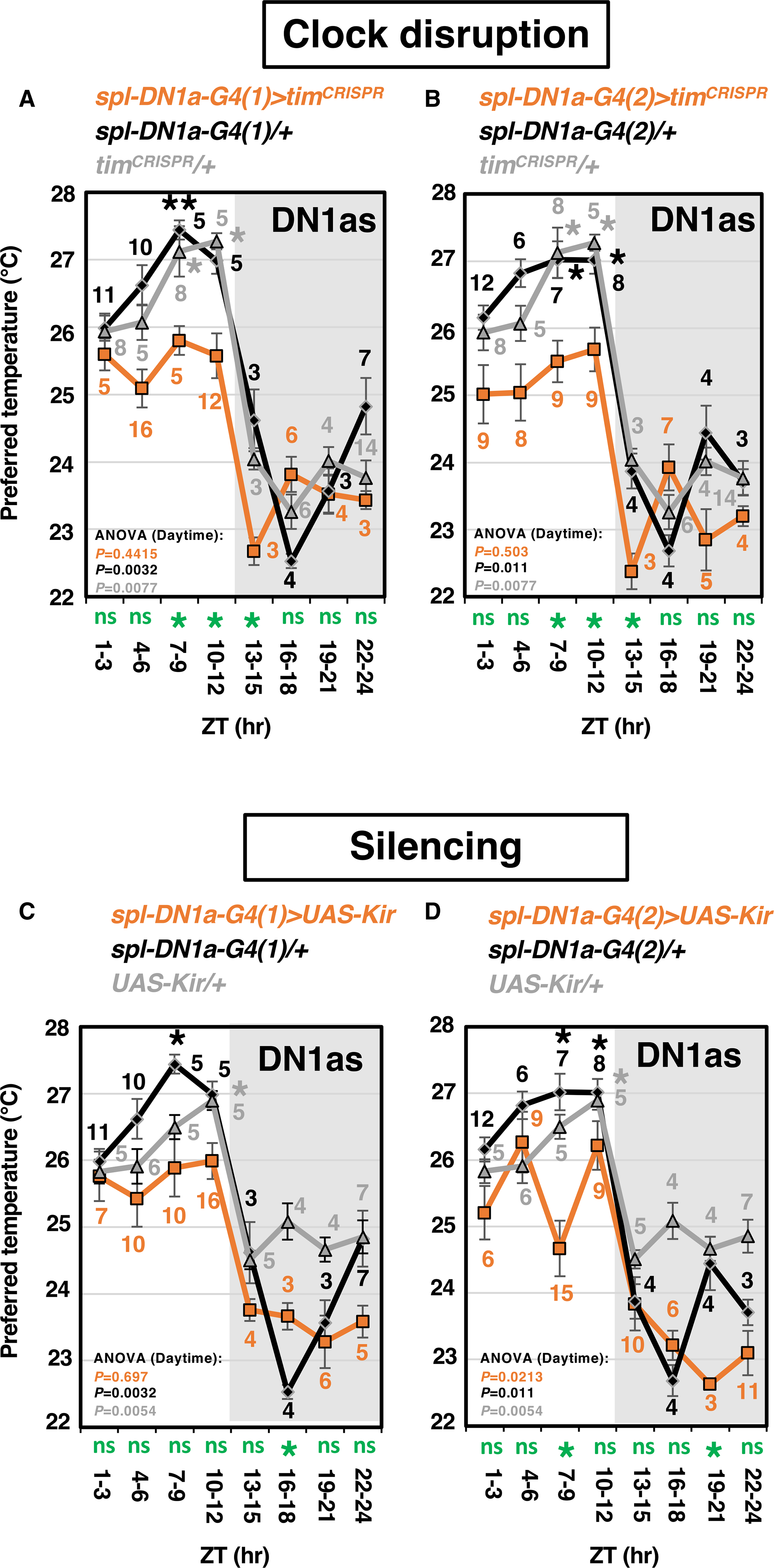
The clock disruption or neural silencing in DN1as causes the abnormal TPR (A–D) The TPR curves observed after clock disruption induced by the expression of *tim*^*CRISPR*^ (A, B) or neuronal silencing induced by the expression of *UAS-Kir2.1* (C, D) in DN1as (orange lines). The following *Gal4* lines targeting DN1as were used: (A, C) *spl-DN1a-G4* (1), (B, D) *spl-DN1a-G4* (2). The TPR data were compared with those at ZT 1–3 using one-way ANOVA and Tukey’s honestly significant difference (HSD) post hoc test (Table S1). The Tp setpoints in experimental and control flies were compared at each time point using one-way ANOVA and Tukey’s HSD post hoc test: green *p < 0.05 for both controls and ns refers to not significant, which are shown below the x axis.

Recent reports suggest that DN1as are the downstream target of cold-processing neurons (Alpert et al., 2020; Marin et al., 2020). We asked whether DN1as are involved in TPR. We found that *tim* knockout in DN1as using either *spl-DN1a-Gal4s (1)* or *(2)* (i.e., *spl-DN1a-Gal4>tim*^*CRISPR*^) abolished the time-dependent Tp increase during the daytime but showed a significant Tp drop at the day-to-night transition (Figures 4A and 4B; Table S1). We verified TIM depletion in DN1as (Figures S2C, S2D, and S2E). Thus, we concluded that clocks in DN1as play important roles in the daytime TPR. Notably, we noticed that DN1as clock-disrupted flies showed a significantly lower Tp than control flies at ZT 7–9, 10–12, and 13–15 (green stars at the bottom of the graph) (Figures 4A and 4B; Table S2). For example, while the Tp in the control flies was around 27°C at ZT 10–12, the Tp in DN1as clock-disrupted flies was around 25.5°C (Figure 4A).

To further examine the role of DN1as in TPR regulation, we expressed *UAS-Kir2.1* in DN1as and tested the TPR. DN1a-silenced flies failed to show the time-dependent Tp increase during the daytime but still exhibited a significant decrease in the Tp at the day-to-night transition (Figures 4C and 4D; Table S1). Therefore, we concluded that DN1as are also important clock neurons for TPR. Taken together, we show that the DNs network plays important role in regulating TPR.

## DISCUSSION

BTR is one of the most conspicuous outputs of the circadian clock and is regulated separately from the locomotor activity rhythm. At least two factors determine the BTR. One is the pattern of body temperature change for 24 h. The other is the setting of the body temperature. However, little is known about how the BTR fluctuation is determined and how animals establish the temperature setpoint. Here, we unveiled in *Drosophila* that the DN networks, the DN2-DN1p microcircuit, and DN1as, determine both the TPR rhythmic pattern and the Tp setpoint.

### The clock functions of DN2s and DN1as in TPR regulation are likely different

We found that the DN2s or DN1as clock-disrupted flies failed to show the time-dependent Tp increase during the daytime (Table S4). The DN2s clock-disrupted flies preferred a similar Tp to the controls during the daytime but a slightly lower Tp than control flies during the nighttime (green ns, Figure 1D; Table S2). However, the DN1as clock-disrupted flies preferred a significantly lower temperature than the controls during ZT 7–15 (Figures 4A and 4B). Therefore, the data suggest that the clocks in DN2s and DN1as have different roles in setting the Tp.

Previously, we showed that the main clock gene, *period* (*per*) null-mutant (*per*^*01*^) flies lost the time-dependent Tp increase during the daytime and that the PER expression in DN2s on *per*^*01*^ flies strongly recovered it (Kaneko et al., 2012). We wonder why the PER expression in DN2s on *per*^*01*^ flies could show a significant time-dependent Tp increase without the clock in DN1as. One possible scenario is that although the rescue flies show the time-dependent Tp increase during the daytime, the TPR curve includes a substantial Tp dip during ZT 7–9 (Kaneko et al., 2012). Therefore, the lack of the clock in DN1as might cause the Tp dip since DN1as clock-disrupted flies show a low Tp setpoint during ZT 7–15 (Figures 4A and 4B; Table S2). Further examinations would be essential to understand the roles of DN2s and DN1as for the TPR mechanisms.

### Temperature inputs via the DN2-DN1p microcircuit and DN1as

How do DN2s and DN1as contribute to the TPR by setting the Tp? Recent papers showed that ACs (warm sensors) (Hamada et al., 2008) project to DN1ps (Jin et al., 2021) and that cold-sensing neurons project posterior antennal lobe (PAL) (Frank et al., 2015) and cold-processing neurons at PAL project to DN1as (Alpert et al., 2020; Marin et al., 2020) (Figure S4). Therefore, the DN2-DN1p microcircuit and DN1as likely receive warm or cold inputs, respectively.

It was shown that DN2s and DN1ps are excited by cooling temperatures (23–16°C) and inhibited by warming temperatures (23–29°C) (Yadlapalli et al., 2018), suggesting that a broader temperature range tunes DN2s and DN1ps. Although DN1as do not respond to cooling (23–16°C) or warming temperatures (23–29°C) (Yadlapalli et al., 2018), DN1as are inhibited by the absolute cold temperature at 20°C (Alpert et al., 2020), suggesting that DN1as may be tuned to a narrower temperature range than DN2s and DN1ps. Thus, the functions of the DN2-DN1p microcircuit and DN1as in setting the Tp are likely different.

We showed that silencing either DN2s or DN1ps lowered the Tp setpoint (Figures 1B, 1C, and 1E; Table S4). DN2- or DN1p-silenced flies cannot avoid lower temperatures, potentially due to the lack of cold sensing; consequently, they remain in a lower-temperature environment. On the other hand, we showed that the silencing of DN1as did not cause significant changes in the Tp compared with the controls (Figures 4C and 4D). We suspect that when DN1as are silenced, another redundant cold-sensing pathway might be activated, and therefore, the flies successfully avoid cold temperatures. Together, the data also support the possibility that the functions of the DN2-DN1p microcircuit and DN1as are likely to separate and are functionally different in setting the Tp.

### The DN2-DN1p microcircuit and Tp setting

DN2-DN1p GRASP contacts show a daily fluctuation, which peaks at the day-to-night transition (Figure 2F). During the daytime (ZT 1–12), the number of DN2-DN1p contacts gradually increases as the Tp also increases. In the evening (ZT 9–12), the number of DN2-DN1p contacts is high when the flies select the highest Tp. The data suggest a potential correlation between the Tp and the number of DN2-DN1p contacts during the daytime. An exception is during the early night, when the DN2-DN1p contacts peak, flies start choosing a lower Tp due to light masking. Therefore, the number of DN2-DN1p contacts might determine the Tp setpoint, particularly during the daytime.

### The role of DN1ps in TPR might incorporate environmental signals and sleep information

What are the functions of DN1ps in TPR regulation? In addition to temperature, we previously showed that DN1ps regulate the light-dependent temperature preference (Head et al., 2015), i.e., a preference for higher temperatures in the presence of light. Because DN1ps are important neurons controlling sleep/wake cycles (Guo et al., 2016, 2017; Kunst et al., 2014; Lamaze et al., 2018; Lamaze and Stanewsky, 2019), DN1ps might be the hub of sleep and TPR, which are inextricably linked. In mammals, sleep and the BTR are closely linked (Krauchi, 2007b; Lack et al., 2008; Weinert, 2010). When the body temperature decreases during the night, the sleep level increases, indicating that sleep and body temperature show a reciprocal relationship at the neural circuit level. Together, the functions of DN1ps might incorporate environmental signals, such as temperature, light, and sleep information into TPR-associated neural circuits (Figure S4).

### Neural circuits for TPR and locomotor activity rhythms are different in the *Drosophila* brain

While the main clock neurons controlling locomotor activity rhythms are LNvs-based circuits, we show here that the main clock neurons controlling TPR are DN-based circuits. We concluded that two circuits controlling the TPR and locomotor activity rhythms are different in the fly brain, although these circuits may partially overlap, are completely segregated, or may be integrated at some levels.

Our data suggest that DNs regulate rhythmic pattern and preferred temperature setting. Thus, a fly TPR analysis would help elucidate the mechanisms of the BTR and provide insights into the relationship between the BTR and locomotor activity rhythm.

### Limitation of the study

We performed and repeated the TPR assays for 30 min using different flies at different times of the day. Therefore, Tp was not continuously monitored for 24 h; instead, individual Tp data obtained using different flies were plotted for 24 h.

Our GRASP results suggest microcircuits from DN2s to DN1ps (Figure 2). Because we used split-GFP components anchored to a membrane protein (CD4) but not to a specific synaptic protein, some GRASP signals are likely extrasynaptic. Nonetheless, since ATP-P2X2 physiological data show the functional connection from DN2s to DN1ps, a portion of the GRASP signal should be contributed by the synaptic contacts from DN2s to DN1ps. Furthermore, our data did not exclude the possibility that DN1ps could still modulate DN2s using another signaling pathway, such as neuropeptide- or non-calcium-based activation. In addition, DN1ps send inhibitory inputs to sLNvs (Fernandez et al., 2020), and sLNvs modulate DN2s (Tang et al., 2017), the activity of DN1ps may indirectly influence DN2s. Therefore, our data do not exclude the possibility of microcircuits from DN1ps to DN2s.

## STAR★METHODS

### RESOURCE AVAILABILITY

#### Lead contact

Further information and requests for resources and reagents should be directed to and will be fulfilled by the lead contact, Fumika Hamada (fnhamada@ucdavis.edu).

#### Materials availability

This study did not generate new unique reagents.

#### Data and code availability

All data reported in this paper will be shared by the lead contact upon request.

This paper does not report original code.

Any additional information required to reanalyze the data reported in this paper is available from the lead contact upon request.

### EXPERIMENTAL MODEL AND SUBJECT DETAILS

All flies were raised on 12-h light/12-h dark cycles at 25°C; Zeitgeber Times (ZT) 0 and 12 refer to the times at which the lights were turned on and off, respectively. All the fly lines used in this study were provided by the Bloomington Drosophila Stock Center, with the exception of the following lines: *UAS-CD4:spGFP1–10* and *LexAop-CD4:spGFP11* (provided by Dr. Kristin Scott), *UAS-P2X2, LexAop-P2X2*, *LexAop-GCaMP* (provided by Dr. Orie Shafer), *UAS-sgRNA-tim*^*3x*^*; UAS-Cas9.2* (provided by Dr. Mimi Shirasu-Hiza), *spl-gDN1-Gal4* (provided by Dr. Gerald M. Rubin) and *UAS-dnATPase* and *UAS-Shaw-RNAi* (provided by Dr. Leslie Griffith). Flies of both sexes were equally used in all experiments unless otherwise noted in the paper.

### METHOD DETAILS

#### Immunohistochemistry

Immunostaining was performed as described previously (Hamada et al., 2008; Tang et al., 2013), with the exception that 5–10% fetal bovine serum in PBST (PBS plus 0.5% Triton X-100) was used for the blocking step and antibody incubations. The following antibodies and dilutions were used: rabbit anti-GFP (1:200; Invitrogen, Cat# A6455; RRID: AB_221570), guinea pig anti-VRI (1:200, from Dr. Hardin), rat anti-TIM (1:200; from Dr. Rosbash), rabbit anti-CCHa1 (1:200; from Dr. Yoshii), rabbit anti-CD4 antibody (1:200; Abcam, Cat# ab133616; RRID: AB_2750883), donkey anti-rabbit-Alexa Fluor 488 (1:200; Jackson IR, Cat# 711–545-152; RRID: AB_2313584), donkey anti-rat-Alexa Fluor 488 (1:200; Jackson IR, Cat# 712–545-153; RRID: AB_2340684), donkey anti-guinea pig-Alexa Fluor 647 (1:200; Jackson IR, Cat# 706–605-148; RRID: AB_2340476) and donkey anti-rabbit-Cy5 (1:200; Jackson IR, Cat# 711–175-152; RRID: AB_2340607). Mounted brains were scanned using a Zeiss LSM5 Pascal confocal microscope, and the images were digitally projected as Z-stacks. The signal intensities of anti-CD4 antibody in DN1ps and anti-GFP antibody in DN2s were measured using ImageJ software and calculated with Excel (Figure 2F).

#### GRASP

The native fluorescence of reconstituted GFP was detected without staining and was specified as overlapping with the Cy5 staining for spGFP1–10 (red) in the target areas to determine the GRASP signal. All brain images were acquired using constant scanning settings to allow comparisons of the fluorescent signals at different times of the day (Figure 3F). Imaris (Bitplane, RRID: SCR_007370) was used to quantify the intensity of all reconstituted GFP signals after background subtraction. We used the intensity to analyze GRASP signals as previously (Tang et al., 2017). In each brain sample, nonclock cell regions next to DN2s were selected for background subtraction. The total intensity of reconstituted GFP signals (GRASP signal) was measured as an indicator of the extent of the DN2-DN1 contacts in the dorsal brain side of these areas.

#### GCaMP imaging

Calcium images were obtained from DN1s in *Clk9M-Gal4;PDF-Gal80::UAS-P2X2*/*4.1M-LexA::LexAop-GCaMP3.0* flies or from DN2s in *Clk9M-Gal4;PDF-Gal80::UAS-GCaMP3.0*/*4.1M-LexA::LexAop-P2X2* flies. The brains were prepared as described previously (Hamada et al., 2008; Tang et al., 2013). The fly brains were dissected in hemolymph-like saline (HL3) consisting of the following components (in mM): 70 NaCl, 5 KCl, 1.5 CaCl_2_, 20 MgCl_2_, 10 NaHCO_3_, 5 trehalose, 115 sucrose, and 5 HEPES, pH 7.1. The prepared brain samples were mounted on a laminar flow perfusion chamber beneath the 40× water immersion objective of a fixed-stage upright microscope (Zeiss Axio Examiner Z1). During the experiments, bath application of ATP was used to activate P2X2-expressing cells. ATP (3 mM) was perfused into the bath solution for ~10 s, and the samples were maintained in the bath until all calcium images were acquired. The fluorescence signal was continuously monitored for at least 1 min after the perfusion of ATP into the bath.

Optical images of the preparations were acquired using a digital CCD camera (C10600–10B-H; Hamamatsu) with a 512 × 512-pixel resolution. The data from each image were digitized and analyzed using AxonVision 4.8.1 (Zeiss). For subsequent analysis, the mean fluorescence intensity of the monitored neuron was calculated for each frame. Concurrently, the background fluorescence (calculated from the average fluorescence of two randomly selected non-GCaMP-expressing areas) was subtracted from the mean fluorescence intensity of the regions of interest in each frame. The background-subtracted values were then reported as the Δ*F*/*F* percentage, where *F* is the mean fluorescence intensity prior to stimulation.

#### Assay of temperature preference behavior and data analysis

All flies were raised at 25°C under LD conditions in the same incubator. The temperature preference assays were not performed continuously for 24 h; instead, they were independently performed for 30 min at different times of the day. The flies used for the behavioral assay were never reused. The temperature preference behavior was performed under light conditions during the daytime and under dark conditions during the nighttime in an environmental room maintained at 25°C and 65–70% relative humidity.

Flies were subjected to a gradient of temperatures ranging from 18 to 32°C to assay their temperature preference behavior. In this setup, ~20–30 flies (both male and female flies) were allowed to select their Tp for 30 min (Hamada et al., 2008). A previously described method was used to calculate the mean Tp (Goda et al., 2014; Kaneko et al., 2012), and polytetrafluoroethylene (Sigma-Aldrich, Cat# 665800) was applied to the cover to prevent flies from staying on the cover. After the 30-min behavioral assay, the number of flies that stayed completely on the apparatus was counted. Flies that were partially or completely on the walls of the apparatus cover were not counted or included in the data analysis. The percentage of flies on the apparatus obtained for each 1-°C temperature interval was calculated by dividing the number of flies within each 1-°C interval by the total number of flies on the apparatus. The location of each 1-°C interval was determined by measuring the temperature at six different points on both the top and bottom of the apparatus. The data points were plotted as the percentages of flies within 1-°C temperature intervals. The weighted mean Tp was calculated by summing the products of the percentage of flies within a 1-°C temperature interval and the corresponding temperature (e.g., fractional number of flies X 18.5°C + fractional number of flies X 19.5°C + … fractional number of flies X 32.5°C). We tested the temperature preference behavior at least five times at each time interval (ZT 1–3, 4–6, 7–9, 10–12, 13–15, 16–18, 19–21 and 22–24). For each of the tested time intervals, the weighted mean Tp obtained from each trial was averaged, and the SEM was calculated.

Because the Tp sometimes varies among different fly lines (Head et al., 2015), the temperature preference of Gal4/UAS flies was always compared with that of Gal4/+ or UAS/+ control flies. Because light affects the temperature preference (Head et al., 2015; Kaneko et al., 2012), the neural circuits associated with the TPR are expected to differ between LD and DD. Therefore, we only focused on LD in this study. The behavioral apparatus and detailed conditions were previously described (Goda et al., 2014).

### QUANTIFICATION AND STATISTICAL ANALYSIS

#### TPR behavior

Statistical comparisons of TPR or the Tp at each time point in experimental and control flies were performed with one-way ANOVA and Tukey’s HSD *post hoc* test using GraphPad Prism7.02. All significance values are denoted in each graph. The numbers of the trials at each time point are shown in the figures.

#### GRASP

The reconstituted GFP signals were analyzed in Imaris to determine the GRASP signal. The GRASP puncta were detected with the spot detection module using identical parameters for all experimental conditions and specified as overlapping with the Cy5 staining for spGFP1–10. The GFP intensity of those puncta was acquired from the statistics tab of the spot function. Graph generation and statistical analyses were done using GraphPad Prism.

#### Calcium Imaging

The physiology package in AxonVision was used to monitor the fluorescence signals and calculate the mean fluorescence intensity of the selected area. After subtraction of the background intensity, ΔF/F percentage plots were generated. The statistical analyses were performed using GraphPad Prism.

## Supplementary Material

1

2

3

4

5

## Figures and Tables

**KEY RESOURCES TABLE:** 

REAGENT or RESOURCE	SOURCE	IDENTIFIER

Antibodies		

Rabbit anti-GFP	Invitrogen	Cat# A-6455; RRID: AB_221570
Guinea pig anti-VRI	Dr. Paul Hardin	
Rat anti-TIM	Dr. Michael Rosbash	
Rabbit anti-CCHa1	Dr. Taishi Yoshii (Fujiwara et al., 2018)	
Rabbit anti-CD4	Abcam	Cat# ab133616; RRID: AB_275088
Donkey anti-rat-Alexa Fluor 488	Jackson IR	Cat# 712-545-153; RRID: AB_2340684
Donkey anti-guinea pig-Alexa Fluor 647	Jackson IR	Cat# 706-605-148; RRID: AB_2340476
Donkey anti-rabbit-Cy5	Jackson IR	Cat# 711-175-152; RRID: AB_2340607

Chemicals, peptides, and recombinant proteins		

HEPES	Sigma	H3375
NaCl	Fisher scientific	BP358-10
KCl	Fisher scientific	BP366-500
CaCl_2_	Sigma	C7902
MgCl_2_	Fisher scientific	BP214-500
NaHCO_3_	Sigma	S5761
Trehalose	Acros organics	182550250
Glucose	Sigma	G6152
Sucrose	Sigma	S0389
Fructose	AMRESCO	0226-2.5KG
Lactose	Sigma	L3625

Experimental models: Organisms/strains		

Drosophila: Clk4.1M-Gal4	Bloomington Drosophila Stock Center (Zhang et al., 2010a; Zhang et al., 2010b)	RRID: BDSC_36316
Drosophila: R18H11-Gal4	Bloomington Drosophila Stock Center (Kunst et al., 2014)	RRID: BDSC_48832
Drosophila: spl-gDN1-Gal4 (R20G07-p65.AD;R18H11-G4.DBD)	Dr. Gerald M. Rubin (Guo et al., 2017)	
Drosophila: UAS-Kir2.1	Dr. Michael Bate (Baines et al., 2001)	
Drosophila: tim^CRISPR^ (UAS-sgRNA-tim^3x^; UAS-Cas9.2)	Dr. Mimi Shirasu-Hiza	
Drosophila: Clk9M-Gal4;PDF-Gal80	Hamada Lab	
Drosophila: UAS-P2X2	Dr. Orie Shafer	
Drosophila: UAS-GCamp3.0	Bloomington Drosophila Stock Center	RRID: BDSC_32116
Drosophila: Clk4.1M-LexA	Bloomington Drosophila Stock Center	RRID: BDSC_80704
Drosophila: LexAop-GCamp3.0	Dr. Orie Shafer	
Drosophila: LexAop-P2X2	Dr. Orie Shafer	
Drosophila: UAS-P2X2	Dr. Orie Shafer	
Drosophila: UAS-dnATPase	Dr. Leslie Griffith	
Drosophila: UAS-Shaw RNAi	Dr. Leslie Griffith	
Drosophila: spl-DN1a-Gal4(1) (VT04317-p65.AD;R93B11-G4.DBD)	Dr. Taishi Yoshii (Sekiguchi et al., 2020)	
Drosophila: spl-DN1a-Gal4(2)	Bloomington Drosophila	RRID: BDSC_70601
R23E05-p65.AD R92H07-Gal4.DBD	Stock Center (Alpert et al., 2020)	RRID: BDSC_70004
Drosophila: UAS-tim RNAi (kk)	Vienna Drosophila Resource Center	101100
Drosophila: LexAop-CD4-spGFP11	Dr. Kristin Scott	
Drosophila: UAS-CD4-spGFP1-10	Dr. Kristin Scott	

Software and algorithms		

ImageJ	National Institutes of Health	RRID: SCR_002285
Imaris	Bitplane	RRID: SCR_007370
AxioVision	ZEISS	RRID: SCR_002677
GraphPad Prism (v 7.02)	Graphpad Software	RRID: SCR_002798

## Highlights

- The DN2-DN1p microcircuit regulates the daytime TPR.
- The silencing of DN2s or DN1ps leads to a lower temperature preference.
- DN1as are the important clock neurons for TPR
- The DN networks control a rhythmic pattern and a temperature setpoint of TPR
