# Supplementary material for: Dorsal clock networks drive temperature preference rhythms in Drosophila
Source: Cell Rep. Author manuscript; Available in PMC 2022 May 16. (PMC9109596; doi:10.1016/j.celrep.2022.110668)
Supplement: 1 [file NIHMS1798656-supplement-1.pdf]

Cell Reports, Volume *39*

Supplemental information

Dorsal clock networks drive temperature preference rhythms in *Drosophila*

Shyh-Chi Chen, Xin Tang, Tadahiro Goda, Yujiro Umezaki, Abigail C. Riley, Manabu Sekiguchi, Taishi Yoshii, and Fumika N. Hamada

Figure S1

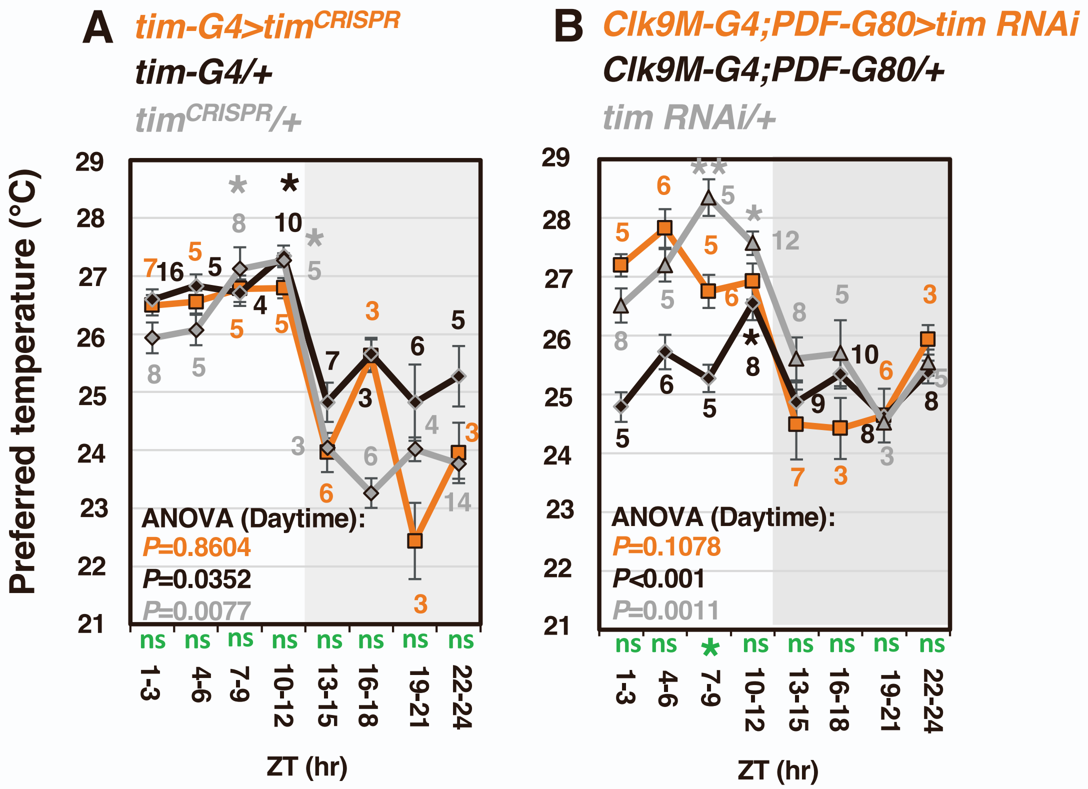
A
tim-G4>tim^CRISPR
tim-G4/+
tim^CRISPR/+
B
Clk9M-G4;PDF-G80>tim RNAi
Clk9M-G4;PDF-G80/+
tim RNAi/+
Preferred temperature (°C)
ANOVA (Daytime):
P=0.8604
P=0.0352
P=0.0077
ANOVA (Daytime):
P=0.1078
P<0.001
P=0.0011
1-3
4-6
7-9
10-12
13-15
16-18
19-21
22-24
ZT (hr)

Figure S1: TIM knockdown causes an abnormal TPR–related to Fig. S1.

TIM depletion and knockdown through the expression of *tim*^{CRISPR} in all clock neurons (A) and *UAS-tim-RNAi* in DN2s (A) led to an abnormal daytime TPR, respectively. Flies with *tim* depletion/knockdown (orange) and control flies (black and gray) are shown. The numbers in the graphs represent the number of assays. The TPR data were compared with those obtained at ZT 1–3 using one-way ANOVA and Tukey's HSD *post hoc* test: $**p < 0.01$, $*p < 0.05$.

Figure S2

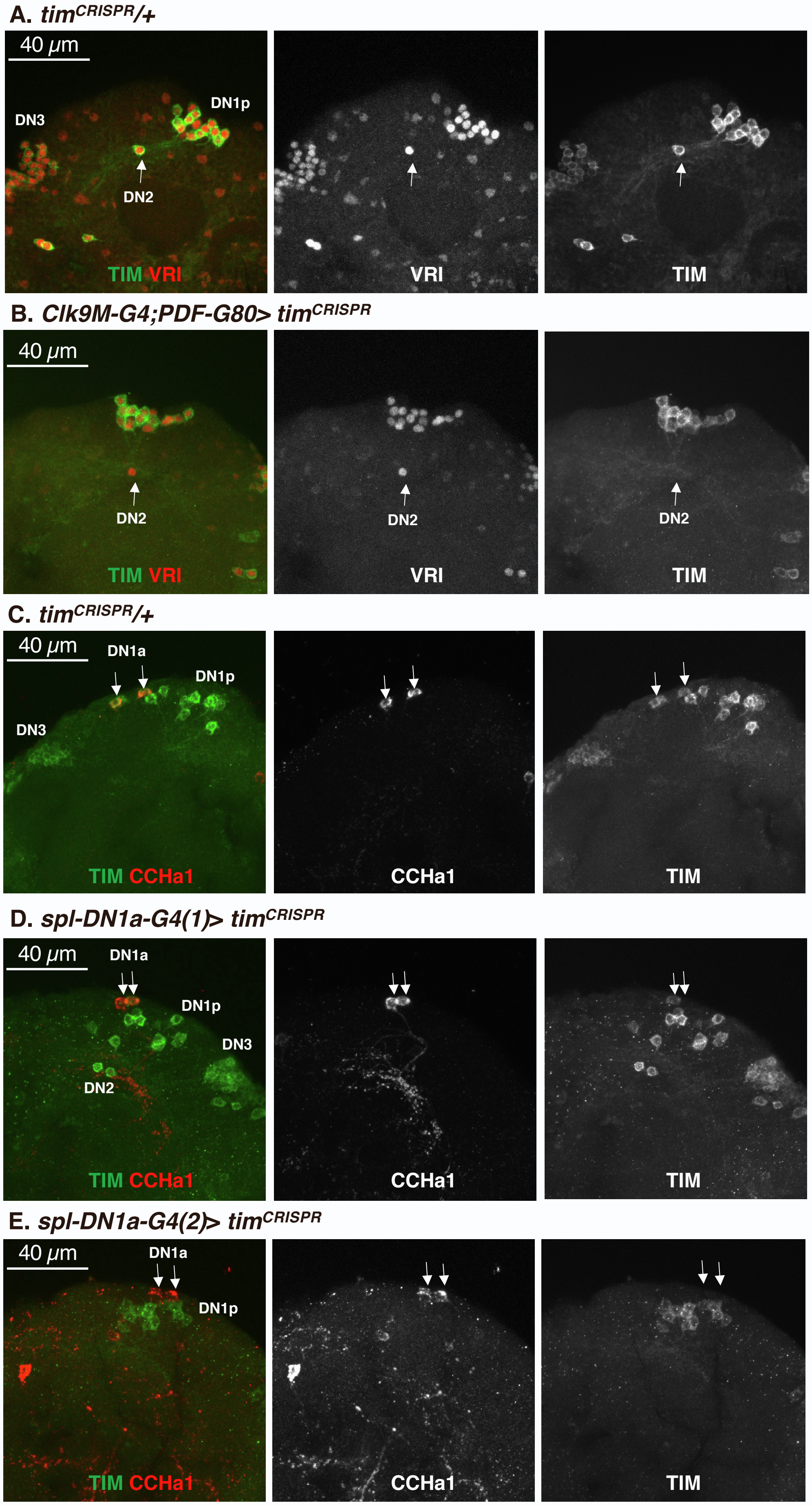
A. timCRISPR/+
40 μm
DN1p
DN3
DN2
TIM VRI
VRI
TIM
B. Clk9M-G4;PDF-G80> timCRISPR
40 μm
DN2
TIM VRI
DN2
VRI
DN2
TIM
C. timCRISPR/+
40 μm
DN1a
DN1p
DN3
TIM CCHa1
CCHa1
TIM
D. spl-DN1a-G4(1)> timCRISPR
40 μm
DN1a
DN1p
DN3
DN2
TIM CCHa1
CCHa1
TIM
E. spl-DN1a-G4(2)> timCRISPR
40 μm
DN1a
DN1p
TIM CCHa1
CCHa1
TIM

Figure S2: Anti-TIM immunostaining in flies expressing *tim*^{CRISPR} in clock neurons; related to Figs. 1 and 4.

The fly brains were immunostained with anti-TIM and anti-VRI antibodies at ZT 16 (**A** and **B**). An anti-VRI antibody was used to label DN2s. TIM protein expression was detected in DN2s in *tim*^{CRISPR}/+ control flies (**A**) but not in *Clk9M-G4;PDF-G80>tim*^{CRISPR} flies (**B**). The fly brains were immunostained with anti-TIM and anti-CCHa1 antibodies at ZT 16 (**C-E**). An anti-CCHa1 antibody was used to label DN1as. TIM protein expression was detected in DN1as in *tim*^{CRISPR}/+ control flies (**C**) but was significantly decreased in both *spl-DN1a-G4(1)>tim*^{CRISPR} (**D**) and *spl-DN1a-G4(2)>tim*^{CRISPR} (**E**) flies.

Figure S3

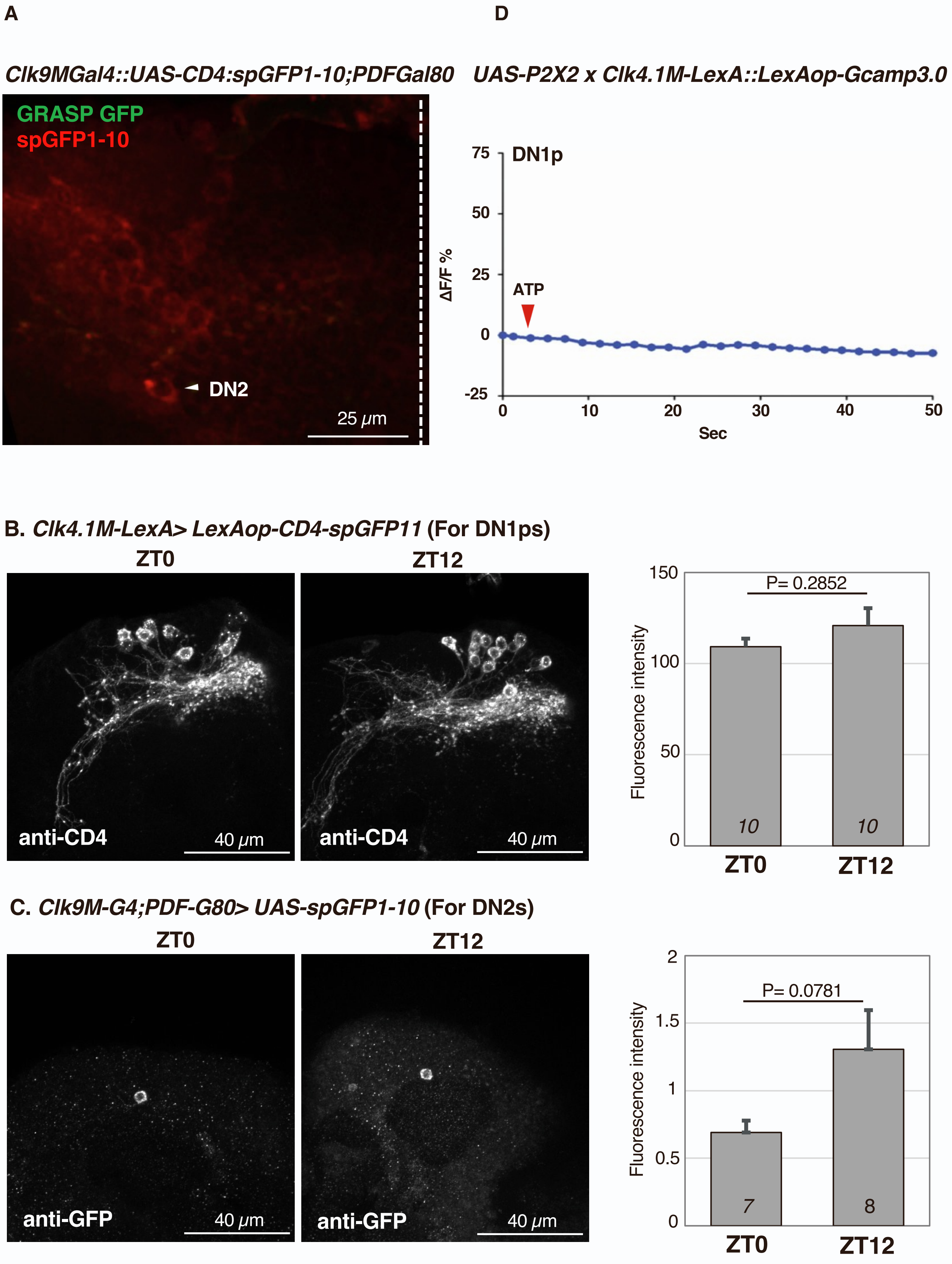

A
D
Clk9MGal4::UAS-CD4:spGFP1-10;PDFGal80
UAS-P2X2 x Clk4.1M-LexA::LexAop-Gcamp3.0
GRASP GFP
spGFP1-10
DN2
25 μm
75
50
25
0
-25
DN1p
ATP
ΔF/F %
0
10
20
30
40
50
Sec
B. Clk4.1M-LexA> LexAop-CD4-spGFP11 (For DN1ps)
ZT0
ZT12
anti-CD4
40 μm
anti-CD4
40 μm
150
100
50
0
P= 0.2852
Fluorescence intensity
10
10
ZT0
ZT12
C. Clk9M-G4;PDF-G80> UAS-spGFP1-10 (For DN2s)
ZT0
ZT12
anti-GFP
40 μm
anti-GFP
40 μm
2
1.5
1
0.5
0
P= 0.0781
Fluorescence intensity
7
8
ZT0
ZT12

Figure S3: GRASP and GCaMP controls–related to Fig. 2

(**A**) Single split-GFP fragment expressed in DN2s of *Clk9M-Gal4::UAS-CD4:spGFP1–10;PDF-Gal80* flies showed no reconstituted GFP fluorescence signals (GRASP GFP; green) in the dorsal brain area around DN2s stained with an antibody against spGFP1–10 (red; shown in arrowhead). The dotted line shows the midline of the brain. **(B and C)** Control experiments for Fig. 3F: The CD4-spGFP11 fusion protein was expressed in DN1ps from the *Clk4.1M-LexA* driver. The expression levels of CD4-spGFP11 were compared between ZT 0 and ZT 12 by staining with an anti-CD4 antibody (**B**)**.** The spGFP1–11 protein was expressed in DN2s from the *Clk9M-G4;PDF-G80* driver. The expression levels of spGFP1–11 were compared between ZT 0 and ZT 12 by staining with an anti-GFP antibody. The italic numbers in the graphs represent the number of brain hemispheres (**C**). In both cases, the difference between ZT 0 and ZT 12 was not statistically significant (t-test: P = 0.2852 (**B**) and P = 0.0781 (**C**)). (**D**) Control experiments for Fig. 3J: A representative graph of DN1p activity without DN2-driven expression of P2X2. GCaMP3.0 was expressed in DN1ps with no P2X2 expression in DN2s using *UAS-P2X2/Clk4.1M-LexA::LexAop-GCaMP3.0* flies. The representative trace of GCaMP fluorescence in DN1ps showed no excitation by ATP.

Figure S4

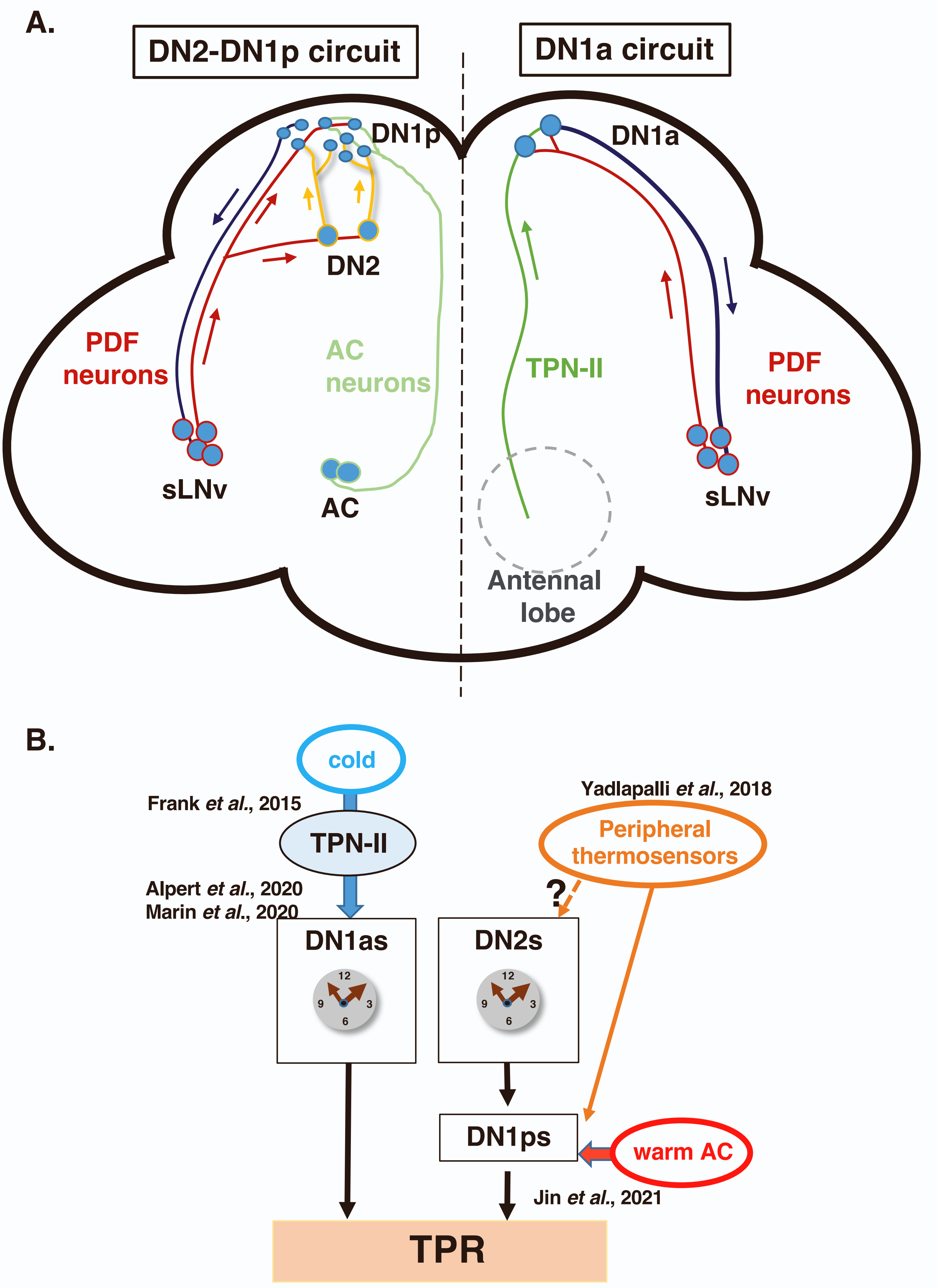
A.
DN2-DN1p circuit
DN1a circuit
DN1p
DN2
PDF
neurons
AC
neurons
sLNv
AC
DN1a
TPN-II
PDF
neurons
sLNv
Antennal
lobe
B.
cold
Frank et al., 2015
TPN-II
Alpert et al., 2020
Marin et al., 2020
Yadlapalli et al., 2018
Peripheral
thermosensors
?
DN1as
12
9
3
6
DN2s
12
9
3
6
DN1ps
warm AC
Jin et al., 2021
TPR

Figure S4: A schematic of the DN2-DN1p microcircuit and DN1a related to Fig. 1,2,3, and 4

(A) A model of the DN2-DN1p microcircuit and DN1as driving TPR. The DN2 circuit includes neuronal connections of DN2s-DN1ps and sLNvs-DN2s (Picot et al., 2009; Tang *et al.*, 2017). Several papers have suggested that the DN1a circuit may include DN1as-TPN-IIs (Alpert *et al.*, 2020) and DN1as-sLNvs (Fujiwara *et al.*, 2018; Shafer et al., 2006). TPN-IIs are thermosensory projection neurons that relay cold temperature information from the posterior antennal lobe (PAL) to DN1as (Alpert *et al.*, 2020). Arrows indicate the direction of neural projections. sLNvs are the main oscillators for locomotor activity rhythms **(B)**. Schematic diagram of the DN2-DN1p microcircuit and DN1as regulating the TPR. The clocks in DN2s and DN1as are responsible for regulating the TPR. For the Tp setting in TPR, cold or warm temperature information may be integrated through DN1as or the DN2-DN1p microcircuit, respectively. ACs: anterior cell neurons.
